# Comparative transcriptomics of a complex of four European pine species

**DOI:** 10.1186/s12864-015-1401-z

**Published:** 2015-03-25

**Authors:** Witold Wachowiak, Urmi Trivedi, Annika Perry, Stephen Cavers

**Affiliations:** Centre for Ecology and Hydrology Edinburgh, Bush Estate, Penicuik, Midlothian, EH26 0QB UK; Institute of Dendrology, Polish Academy of Sciences, Parkowa 5, 62-035 Kórnik, Poland; Edinburgh Genomics, Ashworth Laboratories, University of Edinburgh, Edinburgh, EH9 3JT UK

**Keywords:** Whole transcriptome sequencing, Ontology, SNPs, Nucleotide divergence, Species complex

## Abstract

**Background:**

*Pinus sylvestris*, *P. mugo*, *P. uliginosa* and *P. uncinata* are closely related but phenotypically and ecologically very distinct European pine species providing an excellent study system for analysis of the genetic basis of adaptive variation and speciation. For comparative genomic analysis of the species, transcriptome sequence was generated for 17 samples collected across the European distribution range using Illumina paired-end sequencing technology.

**Results:**

*De novo* transcriptome assembly of a reference sample of *P. sylvestris* contained 40968 unigenes, of which fewer than 0.5% were identified as putative retrotransposon sequences. Based on gene annotation approaches, 19659 contigs were identified and assigned to unique genes covering a broad range of gene ontology categories. About 80% of the reads from each sample were successfully mapped to the reference transcriptome of *P. sylvestris*. Single nucleotide polymorphisms were identified in 22041-24096 of the unigenes providing a set of ~220-262 k SNPs identified for each species. Very similar levels of nucleotide polymorphism were observed across species (π=0.0044-0.0053) and highest pairwise nucleotide divergence (0.006) was found between *P. mugo* and *P. sylvestris* at a common set of unigenes.

**Conclusions:**

The study provides whole transcriptome sequence and a large set of SNPs to advance population and association genetic studies in pines. Our study demonstrates that transcriptome sequencing can be a very useful approach for development of novel genomic resources in species with large and complex genomes.

**Electronic supplementary material:**

The online version of this article (doi:10.1186/s12864-015-1401-z) contains supplementary material, which is available to authorized users.

## Background

Forest trees constitute over 80% of terrestrial biomass and harbour more than 50% of terrestrial biodiversity providing wood material and fundamental ecosystem services for humans including preservation of biodiversity, carbon cycling, climate regulation and preservation of water quality and soils [1,2]. Understanding the genomic basis of adaptation and architecture of complex phenotypic traits is needed for development of diagnostic tools for the conservation, restoration and management of natural populations and for genetic improvement programmes [2]. Understanding plant adaptation is also one of the main interests of evolutionary biology. So far however, knowledge of the mutations, genes and biochemical pathways involved in species evolution and underlying phenotypic and adaptive variation remain scarce mostly due to a lack of efficient methods for accessing the polymorphisms at the whole genome scale. Recent advances in cost-effective, high-throughput sequencing technologies provide new tools for development of genomic resources with huge potential for downstream applications in virtually any species. In particular, these Next-Generation Sequencing (NGS) methods provide a unique opportunity to advance studies of non-model plants, including economically important trees with complex genomes such as conifers [3-5].

Here, we focus on a group of four closely related European pines: Scots pine (*Pinus sylvestris* L.) and the three taxa comprising the *P. mugo* complex including *P. mugo* Turra (dwarf mountain pine), *P. uncinata* Ramond (mountain pine) and *P. uliginosa* Neumann (peat-bog pine). These species differ from each other in phenotype, total population size, geographical distribution and ecology, in particular for traits related to dehydrative stress and temperature [6-8]. *Pinus sylvestris* is one of the most ecologically and economically important forest tree species in the world and has the largest distribution of all pines, being found from western Scotland to eastern Siberia and from Turkey and Spain north to the Arctic Circle. It is locally adapted to environmental conditions related to photoperiod and temperature and shows clinal latitudinal variation in timing of bud set and cold hardiness [9]. *Pinus mugo* is a high-altitude polycormic European pine of up to a few meters in height, which forms shrub populations above the tree line in the mountainous regions of central and southeastern Europe. *Pinus uncinata* and *P. uliginosa* are trees of up to 20 m height; the former is a forest forming component in the high mountains of Western Europe, the latter is adapted to peatbogs in lowland areas of Central Europe.

Despite clear morphological and ecological differentiation, analysis of nuclear genes showed that the species share a similar genetic background, indicating recent divergence [10]. However, despite significant inter- and intra-specific gene flow during historical range shifts, local adaptation to highly contrasting environments has occurred [10,11]. The species are not completely reproductively isolated, can occur and hybridize in sympatry and have the same number of chromosomes (2n = 24). Considering their genetic similarity, but distinctive phenotypes (tree/shrub), geographical ranges (widespread/restricted) and ecology (generalist/specialist) the species comprise a promising system for study of the genomic basis of adaptation and the genetic architecture of phenotypic traits. Taking advantage of the system for comparative studies requires development of a comprehensive array of genomic resources and methods addressing variation at the whole genome scale.

For large and complex genomes, transcriptome sequencing is an attractive alternative to whole genome sequencing, and yields a comparatively high content of functional information from coding regions. By constructing a comparative analysis within a phylogenetic framework we aimed to develop genomic resources relevant to molecular evolution in the genes and gene complexes underlying inter- and intra-specific variation in this important group of tree species.

## Results and discussion

### Characteristics of the transcript sequence

**Figure 1:**
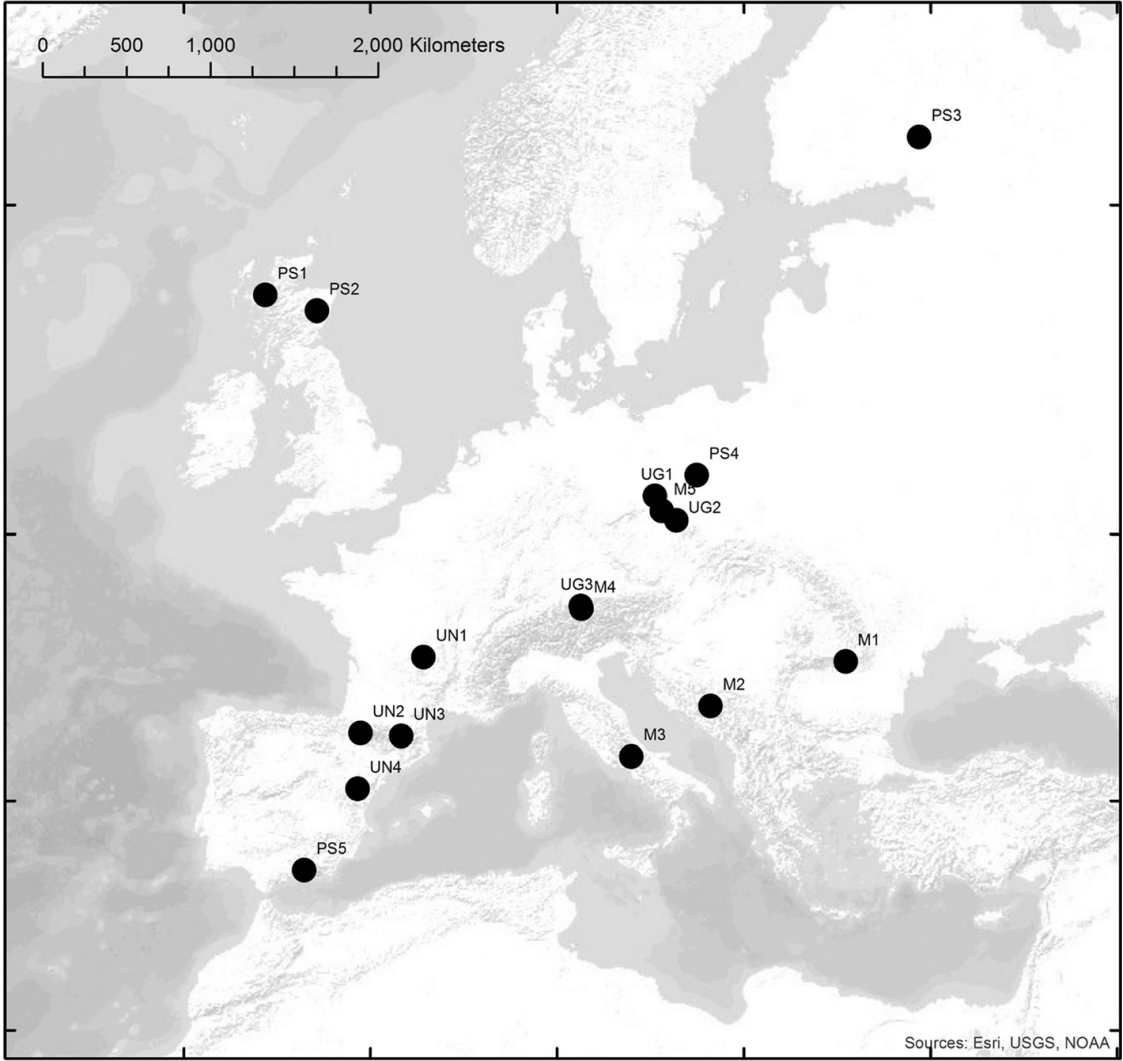
**Locations of the populations of the four pine species sampled for the study.** Populations labelled PS - *P. sylvestris*, M - *P. mugo*, UN - *P. uncinata*, UG - *P. uliginosa*.

Comparative studies of closely related species can advance our understanding of the genetic architecture of adaptive traits. For many species these studies have been seriously limited by a lack of genomic resources from which to develop genetic markers for topics such as species divergence, adaptation and demographic processes in natural populations. In our study we applied Illumina sequencing for successful *de novo* transcriptome characterisation and development of new genomic resources in a complex of four pine species from across the species distribution range in Europe (Figure 1, Table 1). From each insert of the cDNA library, 2 × 100 bp independent reads can be obtained using Illumina paired-end sequencing technology. Our results show that this highly cost and time efficient technology is a very useful and reliable tool for transcriptome characterization, gene discovery and marker development, even for species with large and complex genomes. Sequencing of the reference Scots pine sample (2_GT_31) used for *de novo* transcriptome assembly produced a total of 258,401,512 raw 100 bp sequencing reads. Raw assembly of the reads produced over 151,932 contigs greater than 100 bp that contained over 119 × 10^6^ bp (Table 2). After a series of filtering steps including searches for ORF sequences those contigs were aligned into 40968 unigenes. Retrotransposons comprise a substantial proportion of most plant genomes and they can be transcriptionally active. However, we found less than 0.5% of the unigenes contained such sequences, which is lower than has been found in other plants and pine species. For instance, in the *Pinus contorta* transcriptome, about 6% of contigs represented retrotransposon-like sequences [12]. The low number of retrotransposon sequences may also result from our strict filtering criteria, in which many low quality sequences were discarded before alignment. In our dataset, 170 contigs were identified as putative retrotransposon sequences and they were discarded providing a final set of 40798 high quality unigenes (with mean length of ~1500 bp) and a total reference transcriptome of 61,246,267 bp (Table 2, Additional file 1).

**Table 1:** **Plant material used for transcriptome sequencing**

**Species**	**Acronym**	**Sample ID**	**Population**	**Latitude N**	**Longitude E**	**Altitude (m)**
***P. sylvestris***						
	PS1	1_SD_30	Scotland, Shieldaig	57°30′35″	−5°38′24″	81
	PS2	2_GT_31	Scotland, Glen Tanar	57°2′60″	−2°51′36″	334
	PS3	3_Punk_39	Finland, Punkaharju	61°45′33″	29°23′21″	80
	PS4	4_Jar_43	Poland, Jarocin	51°58′20″	17°28′40″	120
	PS5	5_Trev_37	Spain, Trevenque	37°05′47″	3°32′51″	1170
***P. mugo***						
	M1	6_SC_5	Romania, Southern Carpathians, Busteni	45°25′55″	25°27′06″	2070
	M2	7_BH_9	Bosnia and Herzegovina, Bjelasnica Mts	43°45′00″	18°13′08″	2120
	M3	8_Abr_16	Italy, Abruzzi, La Maiella	41°46′20″	13°58′30″	2200
	M4	9_Alps_12	Austria, Karwendel Mts., Scharnitz	47°22′42″	11°17′45″	1400
	M5	10_Sdt_1	Poland, Sudety Mts, Śląskie Kamienie	50°46′35″	15°36′08″	1400
***P. uncinata***						
	UN1	11_CC_28	France, Col de la Croix de Morand	45°35′58″	2°50′44″	1200
	UN2	12_LaT_23	Spain, Pyrenees, La Trapa	0°32′12″	42°41′19″	1720
	UN3	13_VdR_17	Andorra, Eastern Pyrenees, Vall de Ransol	42°35′02″	1°38′21″	2025
	UN4	14_Val_24	Spain, Sierra de Gudar	40°28′49″	-0°41′51″	2000
***P. uliginosa***						
	UG1	15_Weg_57	Poland, Low Silesian Pinewood, Węgliniec	51°17′50″	15°14′20″	190
	UG2	16_Bat_59	Poland, Wielkie Torfowisko Batorowskie reserve	50°27′32″	16°23′01″	750
	UG3	17_Mit_58	Germany, Mittenwald	47°28′50″	11°16′27″	856

**Table 2:** Statistics for *de novo* transcriptome assembly of the reference sample (2_GT_31)

**Assembly metric**	**Raw assembly generated from Trinity**	**Unigene set**
Max contig length	16652	16652
Num contigs >100	151932	40798
Total bases in contigs >100	119849194	61246267
N50 for contigs >100	1555	2118
Contigs >100 in N50	22593	9640
GC contigs >100	41.8	42.5
nonATGC in contigs >100	0	0
Mean length for contigs >100	788.8	1501.2

N50 - the contig length for which the collection of all contigs of equal or longer length produces half the bases of the contigs.

Non ATGC - non ATGC bases (such as Ns).

**Figure 2:**
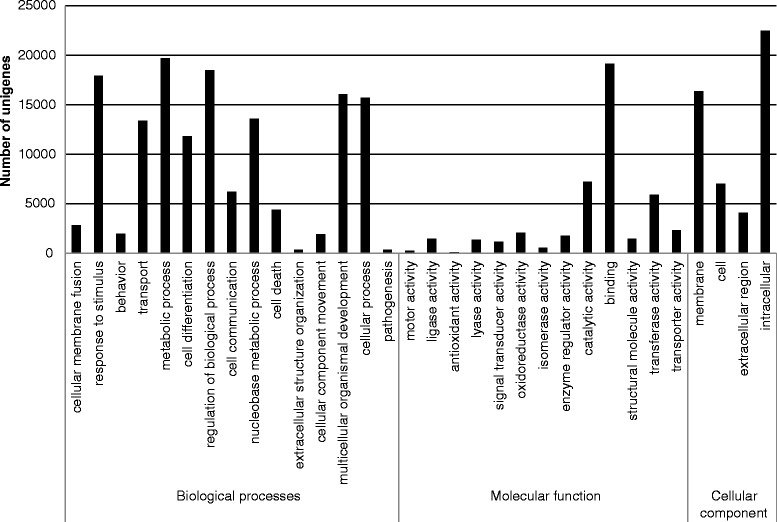
**Gene ontology classification of the unigenes.**

Lack of a reference genome prevented us from estimating the number of genes and transcript coverage for the focal species. However, 48% of the unigenes matched known proteins, providing large set of target genes representing various metabolic pathways. The functions of unigenes covered a broad range of gene ontology categories that were assigned to 19659 unique genes with BLAST matches to known proteins. There were a total of 13653 gene ontology terms associated with those genes. Based on the Kyoto Encyclopedia of Genes and Genomes (KEGG) Pathway 12387 unigenes (~30%) had significant matches in the database and were assigned to 304 pathways. 9529 of the unigenes that had enzyme commission (EC) numbers were assigned to 2130 enzyme pathways. Based on biological processes the most numerous contigs were classified as related to metabolism (19727) and regulation of biological processes (18483). The function of about 43% of the unigenes was related to binding activity. About 78% of all unigenes were classified as intracellular or membrane components (Figure 2, Additional file 1: Table S1). The number of assigned contigs was similar to studies in *P. contorta*, when about 17000 unique genes were found across 63657 contigs developed using a 454 GS XLR70 Titanium pyrosequencer [12]. From the published gene numbers for *Pinus taeda* (~50,000 genes [3]) and other conifer species [4,5] we estimate that we have identified around half of the total number of Scots pine genes. Considering that the focal species are known to be highly diverged for adaptive traits, polymorphisms in the genes belonging to metabolic and regulation pathways are likely to be particularly useful for searching for the genetic basis of quantitative trait variation and local adaptation.

### Marker development

The focal pine species are evolutionarily closely related [10,13] but differ in ecology, geographical distribution and population size. Therefore, they form a very attractive model for studies of the genetic basis of local adaptation and speciation. So far, genetic studies of the species (mostly *Pinus sylvestris*) have focused on assessments of quantitative trait variation and underlying QTLs [14], genetic structure, demography and selection [15-18]. These studies were mostly based on microsatellite loci and/or sequence variation at candidate genes and consequently their conclusions were limited by the low number and resolution of markers or genomic regions. Several QTLs for phenology and polymorphism due to natural selection at a few candidate genes related to stress response were found for Scots pine [14,16,17]. Recent studies have also provided nucleotide polymorphism information for *P. mugo* based on amplicon sequencing and candidate gene studies [6,19]. However, no genomic resources currently exist for this group of pine species to address fundamental questions about the genetic basis of adaptation and divergence. Our study makes a large proportion of the functional variation in coding regions of the genome available for downstream research with the use of high throughput genotyping platforms. In our dataset, the 16 samples of the four pine species sequenced in lower depth produced a total of ~714 × 10^6^ reads with their number varying between 30–69 × 10^6^ per sample (Table 3). The vast majority of all reads for each sample (about 80%) were successfully mapped to the reference transcriptome sequence of the Scottish *Pinus sylvestris* sample from Glen Tanar (2_GT_31 sample). Compared to the reference, from ~64 × 10^3^ (*P. sylvestris* from Finland) to ~148 × 10^3^ SNPs (*P. uliginosa* from Germany) were called for each sample (Table 3). SNPs were found in 54-59% of all unigenes including 22041 unigenes with SNPs identified for *P. sylvestris*, 24096 for *P. mugo*, 22416 for *P. uncinata* and 22710 for *P. uliginosa*. Filtering of all available SNPs from merged contigs across the species that were at least 50 bp apart from each other provided a set of 259,087 SNPs (Additional file 1). The availability of cost and time efficient genotyping methods for SNPs using next-generation sequencing platforms will certainly advance comparative genomic and population genetic studies of these species. The resources could also be useful in breeding and silviculture, through marker-assisted and genomic selection approaches [20], for genetic improvement of phenotypic traits of economic and ecological importance, especially in Scots pine.

**Table 3:** **Mapping statistics of the samples to the reference transcriptome sequence (2_GT_31)**

**Sample ID**	**Species**	**Total reads**	**Mapped reads**	**% Mapped reads**	**% Duplicate reads**	**% Mapped reads as proper pairs**	**Number of SNPs**
1_SD_30	*P. sylvestris*	31116472	26922787	86.52	26.36	84.14	67817
2_GT_31	*P. sylvestris*	258401512	229042493	88.64	43.22	85.34	81519
3_Punk_39	*P. sylvestris*	37849782	31980676	84.49	34.37	80.21	63874
4_Jar_43	*P. sylvestris*	38970706	31951845	81.99	19.34	79.47	94021
5_Trev_37	*P. sylvestris*	45140044	38150182	84.52	19.44	81.73	95814
6_SC_5	*P. mugo*	43752078	35804512	81.83	24.08	78.50	116762
7_BH_9	*P. mugo*	32600000	26565484	81.49	26.23	78.40	103818
8_Abr_16	*P. mugo*	40104880	33111153	82.56	29.18	78.19	100602
9_Alps_12	*P. mugo*	52934684	43411825	82.01	23.36	78.67	130942
10_Sdt_1	*P. mugo*	69248828	57560565	83.12	28.02	80.03	138989
11_CC_28	*P. uncinata*	34805254	28627271	82.25	16.74	79.34	102037
12_LaT_23	*P. uncinata*	30291214	24678075	81.47	36.80	78.06	87181
13_VdR_17	*P. uncinata*	45834740	36942040	80.60	18.27	77.85	118800
14_Val_24	*P. uncinata*	48550644	39819426	82.02	21.98	79.12	115050
15_Weg_57	*P. uliginosa*	52718596	42948249	81.47	18.33	78.55	127068
16_Bat_59	*P. uliginosa*	40729720	33913094	83.26	21.04	80.38	116297
17_Mit_58	*P. uliginosa*	69457322	57212028	82.37	22.07	79.37	147646
**Merged**	**All**	**976529136**	**817402576**	**83.70**	**26.12**	**80.58**	**164104**

Sample ID with reference to Table 1.

### Nucleotide polymorphism and genetic relationships between species

**Figure 3:**
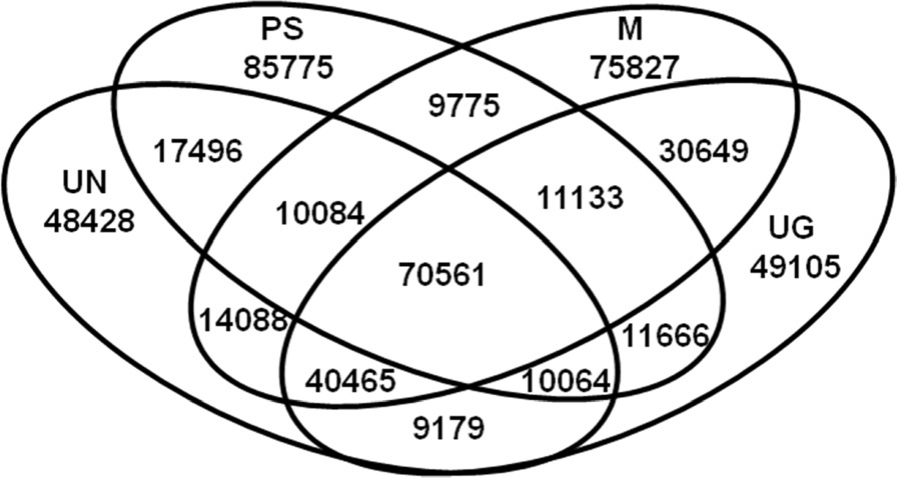
**Shared and unique SNPs in pairwise comparisons between** ***P. sylvestris*** **(S),** ***P. mugo*** **(M),** ***P. uncinata*** **(UN) and** ***P. uliginosa*** **(UG).** SNPs total: *P. sylvestris* (225544), *P. mugo* (262582), *P. uncinata* (220365), *P. uliginosa* (232822).

**Figure 4:**
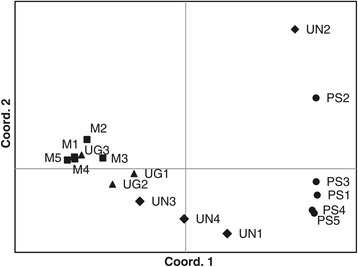
**Principal Coordinates Analysis (PCoA) based on pairwise nucleotide difference matrix at 676 contigs (>1.3Mbp, 27929 SNPs) showing genetic relationships between** ***P. sylvestris*** **(●),** ***P. mugo*** **(■),** ***P. uncinata*** **(♦) and** ***P. uliginosa*** **(▲) samples.**

The samples from which transcriptome data were generated were collected across broad environmental gradients, throughout the species distribution range. Despite clear differences in range and total population sizes, we observed very similar levels of nucleotide polymorphism in each species. Comparing among species, and across the whole transcriptome, most SNPs were found in *P. mugo* (~295 × 10^3^) relative to the reference. This species showed much greater similarity to the other two taxa from the *P. mugo* complex than to *P. sylvestris*, as evident from the higher proportion of common (~144-163 × 10^3^) and lower proportion of unique SNPs (~93-145 × 10^3^) between the *P. mugo* complex taxa as compared to *P. sylvestris* (~65-69 × 10^3^ and ~190-230 × 10^3^, respectively) (Table 4, Figure 3). All four species showed similar levels of nucleotide polymorphism (π_tot_ = 0.0044-0.0053) and an excess of low frequency variation (D = ~ −0.2) (Table 5). Our estimates of total nucleotide polymorphism were very similar to estimates obtained from much smaller candidate gene datasets [6,16-18]. In our study nearly half of the transcriptome sequences were monomorphic across species. Overall, the species showed a high level of genetic similarity marked by similar proportions of reads from different species that mapped to the reference Scots pine transcriptome and many shared SNPs segregating between species. Our study provides evidence for closer genetic relationships between *P. mugo* and *P. uliginosa* as compared to *P. sylvestris* (Additional file 1: Figure S1). *Pinus uncinata* also showed a closer relationship to the taxa from the *P. mugo* complex (Figure 4, Additional file 1: Table S2) except for one outlier sample from Spain that showed closer genetic similarity to *P. sylvestris*. This individual may represent an admixed genotype of both species as cryptic hybrids between *P. uncinata* and *P. sylvestris* were described in Spain in morphological and molecular studies [11]. No significant genetic differentiation (p < 0.05) was found between *P. mugo* and *P. uncinata* vs. *P. uliginosa* (Table 6). Our results are in line with previous evolutionary assessments in these species that showed high genetic identity between the taxa from the *P. mugo* complex and outgroup Scots pine. The close genetic similarity between taxa (especially in the *P. mugo* complex) but high divergence makes them a very promising system for comparative genomic studies. Searches for loci of high divergence against the genetic background of the focal taxa will help to identify regions under selection, which have played a role in adaptation and speciation.

**Table 4:** **Common and unique SNPs in pair-wise comparisons between species**

**Whole transcriptome**				
**COMMON SNPs**				
	***P. sylv.***	***P. mugo***	***P. uncin.***	***P. ulig.***
***P. sylvestris***	**119387**			
***P. mugo***	65646	**294958**		
***P. uncinata***	69345	149679	**246367**	
***P. uliginosa***	65390	162769	144422	**255447**
**UNIQUE SNPs (in reference to the species in each column)**				
***P. sylvestris***		229312	189735	190084
***P. mugo***	53728		109363	92675
***P. uncinata***	50042	145279		111026
***P. uliginosa***	53997	132189	114644	

Total number of SNPs within each species is marked in bold.

**Table 5:** **Nucleotide variation at 676 merged nuclear (** ***n*** **DNA) contigs in the pine species**

**Species**	**N**	**L**	**SNPs**	**Sing.**	**π**_**tot**_	**D**
*P. sylvestris*	5	1364676	12920	8674	0.0044	−0.243
*P. mugo*	5	1364676	13129	8710	0.0045	−0.221
*P. uncinata*	4	1364676	13420	10374	0.0053	−0.169
*P. uliginosa*	3	1364676	9581	9581	0.0047	-
**Total/Aver.**	**17**	**1364676**	**27929**	**12181**	**0.0047**	**−0.211**

N- number of samples analysed; L – length of the sequences in base pairs; SNPs- number of polymorphic sites detected; Sing – number of singleton mutations; π_tot_ – total nucleotide diversity (Nei [31]); D – multilocus Tajima’s D statistics [32].

**Table 6:** **Pairwise** ***Fst*** **between species at 27929 SNPs identified at 676 merged nuclear (** ***n*** **DNA) contigs**

	***P. sylvestris***	***P. mugo***	***P. uncinata***
*P. mugo*	0.257**		
*P. uncinata*	0.142*	0.121*	
*P. uliginosa*	0.212*	0.030	0.075

Significance level: * p < 0.05, **p < 0.01.

## Conclusions

1. We provide a reference transcriptome sequence for Scots pine, a conifer tree species of great ecological and economic importance in the world. We annotated the transcriptome in reference to many genes and metabolic pathways described in open access databases.
2. Putting our study in a phylogenetic framework we provide novel genomic resources comprising a publicly-available database of SNP markers for a set of four closely related pine species. Information about nucleotide polymorphism in coding regions will facilitate genotyping, population genetic and association studies to better understand the genetic basis of plant adaptation and speciation.
3. Our study shows the largest genetic divergence between *P. mugo* and *P. sylvestris*. Despite large differences in distribution range and total population size, all species showed very similar patterns of nucleotide polymorphism.
4. Our results demonstrate the high relevance of Illumina technology for *de novo* assembly, transcriptome characterization and marker discovery in a species with large and complex genomes, which lack draft genome sequence information.

## Methods

### Plant material and RNA extraction

Needles of the four pine species were collected from two year old seedlings grown in a glasshouse at the Centre for Ecology and Hydrology, Edinburgh, UK. The seedlings were obtained from seeds collected in seventeen populations of the species (five for each of *P. sylvestris* and *P. mugo*, four for *P. uncinata* and three for *P. uliginosa*) from across the species distribution range and environmental gradients in Europe (Table 1, Figure 1). After sampling, the needles were immediately frozen in liquid nitrogen and homogenized with a pestle and mortar. Total RNA for generation of transcript sequence was extracted from 100 mg of the needle powder using Spectrum™ Plant Total RNA Kit (Sigma) following the manufacturer’s protocol. RNA concentration and quality was assessed with the use of a Qubit® Fluorometer (Life Technologies). A total of 10 μg of input RNA for each sample was used for normalized cDNA library preparation.

### cDNA library construction and sequencing

Template cDNA libraries for each sample were prepared using TruSeq™ RNA Sample Preparation Kits (Illumina). The poly‐A containing mRNA molecules were purified in two steps from 10 μg of total RNA using poly‐T oligo‐attached magnetic beads. During the second elution of the poly‐A RNA, the RNA was fragmented to 120-210 bp inserts (by incubation of the samples at 94°C for 8 minutes) and primed for cDNA synthesis. The cleaved RNA fragments primed with random hexamers were reverse transcribed into first strand cDNA followed by DNA Polymerase I second strand cDNA synthesis and RNase H treatment. Ampure XP beads were used to separate the double strand cDNA from the 2nd strand reaction mix. The synthesized cDNA was subjected to end-repair to convert the overhangs resulting from fragmentation into blunt ends. These repaired cDNA fragments were adenylated at 3′ ends to prevent them from ligating to one another during the adapter ligation reaction. Paired-end adapters were ligated to the ends of these double strand cDNA preparing them for hybridization onto a flow cell. DNA fragments that had adapter molecules on both ends were enriched by PCR to amplify the amount of DNA in the final cDNA library. Normalization of cDNA was conducted to increase the chance of discovering genes of low expression level. Quality control of the sample libraries and quantification of the DNA templates was conducted using Agilent Technologies 2100 Bioanalyzer using Agilent DNA1000 chip. The cDNA libraries were sequenced using Illumina HiSeq 2000 platform at Edinburgh Genomics, the University of Edinburgh, Scotland according to the manufacturer’s instructions (Illumina, San Diego, CA). Sequencing was conducted to generate 100 base paired-end reads for all samples including the Scots pine sample (2_GT_31, Scotland, Glen Tanar) used as a reference. Raw data for all samples were deposited in European Nucleotide Archive [ENA accession number: PRJEB6877].

### Reference transcriptome assembly and gene annotation

Prior to assembly, filtering of the raw reads for the reference sample 2_GT_31 was carried out to increase the quality of data and eliminate any sequencing errors. Reads with adapter contamination, potential contaminant, and poor-quality reads with ambiguous sequences “N” were discarded. Reads were *de novo* assembled into contigs using Trinity (version r2012-06-08) [21]. We got 151932 potential transcripts as an output. In order to reduce the redundancy in this dataset, only transcripts with ORFs were retained, and highly similar sequences were clustered (similarity level of >95%) using CD-HIT [22]. A final set of 40968 clustered transcripts was BLASTx scanned for the presence of known retrotransposons and repetitive elements known to be present in conifer genome. Several search approaches were used including queries of known retrotransposon sequences in plants (IFG7, GYMNY, *PtIFG7,* Ta1-3, PpRT1) and searches for terms associated with retroelements such as copia, gypsy, gag, retrotransposon, integrase, retroelement, reverse transcriptase [23-25]. Using the above approaches 170 contigs were identified that may represent transcriptionally active retroelements. They were excluded from final reference transcriptome sequences of 40798 contigs. Annotation of the clustered transcripts based on the functional category was conducted using Annot8r based on BLAST similarity searches against annotated subsets of EMBL UniProt protein sequence and functional information database using an *E-value* threshold of 10^−5^ [26]. BLASTx search was conducted against the Kyoto Encyclopedia of Genes and Genomes (KEGG) Pathway with an *E-value* cutoff of <10^−5^ to annotate the genes to known proteins and to look at the networks of molecular functions and interactions of the unigenes. Gene Ontology (GO) classification of the unigenes based on BLAST matches to known proteins was conducted based on biological processes, molecular function and cellular component.

### Alignment, SNP calling and filtering

The set of 40798 transcripts (2_GT_31) was used as reference for mapping reads for the 16 other samples. Alignment was performed using BWA (version 0.6.1) [27]. Duplicates were marked using Picard [28]. To eliminate errors due to indel misalignment, local realignment was conducted using GATK [29]. SNPs were called for each sample and species as compared to the reference using Samtools (version samtools-0.1.18) [28]. A set of SNPs identified across all samples was filtered to look for those suitable for genotyping platforms such as Illumina with a minimum spacing between SNPs of 50 base-pairs (bp) flanking nucleotides on either side of a SNP.

### Nucleotide polymorphisms and divergence

Polymorphism and divergence were quantified within and among species to provide information about the overall pattern of nucleotide variation in the samples. The number of shared and unique SNPs was calculated based on calls from pairwise comparisons between each species. A subset of contigs were selected that were common to, and polymorphic in, all samples relative to the reference. Fasta files for each contig were produced using vcf-tools [30] and concatenated into a single sequence for each sample. In total 1,364,676 bp of DNA was aligned across 676 contigs. Basic statistics including number of polymorphic sites, nucleotide diversity (measured as the average number of nucleotide differences per site (π) between two sequences [31]) and divergence between species were estimated using DnaSP v.5 [33]. Relationships between samples were assessed using Principal Coordinate Analysis (PCoA) based on a pairwise genetic distance matrix (number of base differences per sequence) between samples, and using the UPGMA method based on the number of substitutions per site from averaging over all sequence pairs between groups using the Tamura-Nei model [34]. Polymorphism at the common set of 676 merged nuclear contigs was used to evaluate the genetic differentiation in pairwise comparisons between species. Significance was estimated by 1000 permutations of the samples between species using Arlequin v.3.5 [35]. The outlier *Pinus uncinata* sample (UN2), defined based on PCoA analysis, was excluded from divergence estimates in UPGMA and the species genetic differentiation analysis.

### Supporting data

The datasets supporting the results of this article are freely available through the NERC’s Environmental Information Data Centre, as follows:

1. The sequence of 40798 transcripts of the reference Scots pine sample (2_GT_31):
  Filename: Reference_PS2_trinity.fasta; URL: http://doi.org/10.5285/b6900166-ded6-4f7a-8734-484b6f77b2f1
2. SNP files for each sample with reference to Scots pine transcriptome sequence (2_GT_31):
  Filenames: PS1_SNPs.vcf; PS2_SNPs.vcf; PS3_SNPs.vcf; PS4_SNPs.vcf; PS5_SNPs.vcf; M1_SNPs.vcf;
  M2_SNPs.vcf; M3_SNPs.vcf; M4_SNPs.vcf; M5_SNPs.vcf; UN1_SNPs.vcf; UN2_SNPs.vcf;
  UN3_SNPs.vcf; UN4_SNPs.vcf; UG1_SNPs.vcf; UG2_SNPs.vcf; UG3_SNPs.vcf;
  URL: http://doi.org/10.5285/b6900166-ded6-4f7a-8734-484b6f77b2f1

## Additional file

Additional file 1: Table S1. Gene ontology classification of the unigenes based on biological processes, molecular function and cellular component. **Table S2.** Pairwise nucleotide divergence between species. **Figure S1.** Relationships between species based on pairwise genetic distance at 676 unigenes (27929 SNPs). Outlier *P. uncinata* sample (UN2) was excluded from the analysis.
