# Supplementary material for: Comparative transcriptomics of a complex of four European pine species
Source: BMC Genomics. 2015 Mar 25;16(1):234. doi: 10.1186/s12864-015-1401-z (PMC4458023; doi:10.1186/s12864-015-1401-z)

SUPPLEMENTARY MATERIAL

COMPARATIVE TRANSCRIPTOMICS OF A COMPLEX OF FOUR EUROPEAN PINE SPECIES

Witold Wachowiak, Urmi Trivedi, Annika Perry, Stephen Cavers

Supplementary Table 1. Gene ontology classification of the unigenes based on biological processes, molecular function and cellular component.

**Biological processes**		**Molecular function**		**Cellular component**	
cellular membrane fusion	2863	motor activity	270	membrane	16387
response to stimulus	17946	ligase activity	1472	cell	7043
behavior	1960	antioxidant activity	96	extracellular region	4118
transport	13394	lyase activity	1381	intracellular	22484
metabolic process	19721	signal transducer activity	1185		
cell differentiation	11843	oxidoreductase activity	2053		
regulation of biological process	18483	isomerase activity	567		
cell communication	6243	enzyme regulator activity	1802		
nucleobase metabolic process	13606	catalytic activity	7240		
cell death	4405	binding	19128		
extracellular structure organization	365	structural molecule activity	1458		
cellular component movement	1924	transferase activity	5925		
multicellular organismal development	16094	transporter activity	2347		
cellular process	15727				
pathogenesis	349				

Supplementary Table 2. Pairwise nucleotide divergence between species.

	PS	M	UN
M	0.0060		
UN	0.0053	0.0055	
UG	0.0058	0.0047	0.0053

Supplementary Figure 1. Relationships between species based on pairwise genetic distance at 676 unigenes (27929 SNPs). Outlier *P. uncinata* sample (UN2) was excluded from the analysis.

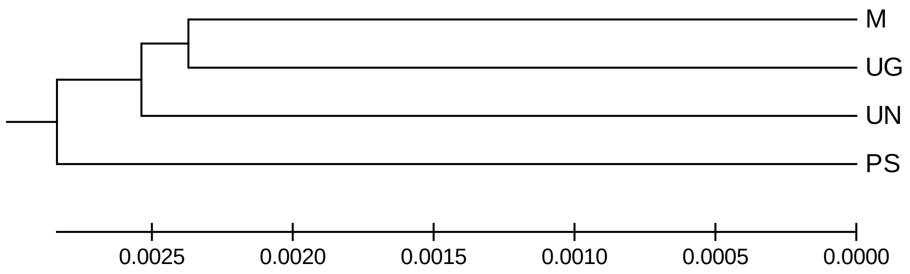

Supplement: Additional file 1: Table S1. — Gene ontology classification of the unigenes based on biological processes, molecular function and cellular component. Table S2. Pairwise nucleotide divergence between species. Figure S1. Relationships between species based on pairwise genetic distance at 676 unigenes (27929 SNPs). Outlier P. uncinata sample (UN2) was excluded from the analysis. [file 12864_2015_1401_MOESM1_ESM.pdf]
